# Focal Segmental Glomerulosclerosis Preceding Type 2 Papillary Renal Cell Carcinoma

**DOI:** 10.1155/2020/8811905

**Published:** 2020-10-15

**Authors:** Dominik Dabrowski, Ekin Ozluk, Silvia Barbeito, Eric X. Wei

**Affiliations:** ^1^Department of Pathology and Translational Pathobiology, LSU Health Shreveport, USA; ^2^Department of Radiology, LSU Health Shreveport, USA

## Abstract

Renal cell carcinoma (RCC) is the predominant renal malignancy in adults. Of the four general subtypes, papillary renal cell carcinoma (P-RCC) is the second most common and can be subdivided into type I, type II, and a mixture of type I and II. Focal segmental glomerulosclerosis (FSGS) is the most common glomerulopathy at all ages, and it can be seen as a paraneoplastic syndrome. RCC, in general, is known to present with many paraneoplastic syndromes, and glomerulopathies are among these. Rarely, RCC and glomerulopathies may overlap in the same patient. Here, we report a 58-year-old male with a past medical history of FSGS and chronic kidney disease (CKD), stage III, who was found to have an incidental renal mass that was later diagnosed as type II P-RCC. The histology showed pseudostratified tumor cells with an eosinophilic cytoplasm that formed papillary configurations and displayed areas of necrosis. The prior FSGS diagnosis exhibited segmental sclerosis, refractory tufts, and capillary membrane wrinkling. A period of 1.5 years elapsed between the diagnosis of the glomerulopathy and the malignancy. The tumor was found to be at stage TIb. To our knowledge, this may be the first reported case of usual-type FSGS as paraneoplastic glomerulopathy (PG) preceding P-RCC. Because FSGS only sparingly affects the kidney and is a common glomerulopathy in adults, it is reasonable to complete comprehensive diagnostic studies and commence medically necessary treatment, especially in the background of other renal comorbidities. These preexisting comorbidities may be associated with malignancy very early in its course. The probability of RCC-associated paraneoplastic glomerulopathy is low, which means an already incidentally found renal mass may conceal a serpentine paraneoplastic syndrome. A more developed understanding of these manifestations can lead experienced clinicians to suspect and possibly uncover an insidious RCC before it advances.

## 1. Introduction

Renal cell carcinoma (RCC) is the most common renal malignancy in adults. RCC can be divided into four general subtypes: clear cell (conventional), papillary (chromophilic), chromophobe, and clear cell papillary, among other rare subcategories. After clear cell RCC, the most common is papillary RCC (P-RCC), which can be further subdivided into type 1, type 2, and mixed type 1 and 2. Type 1 is distinct from type 2 in that the papillae tend to form a single layer of cells and are more basophilic in appearance, whereas type 2 is prone to have a pseudostratified cell layer with significantly more cytoplasm than type 1 and also contrasts as more eosinophilic. Either type may be variably necrotic, although type 2 is more likely to demonstrate necrosis. While many RCCs are found incidentally on imaging, including the current case, a careful clinical history taking can reveal clues to future diagnosis, a year or more, before it is confirmed. Here, we report a case of a 58-year-old male with type 2 P-RCC who presented with focal segmental glomerulosclerosis (FSGS) and chronic kidney disease (CKD) on an earlier admission, 1.5 years before the eventual diagnosis of malignancy. This suggests that FSGS may be a paraneoplastic glomerulopathy (PG) associated with P-RCC. Rather than viewing them as distinct entities, common renal comorbidities, such as hypertensive nephropathy, glomerulopathy, later presentation as chronic kidney disease, and eventual renal malignancy, may be a continuum in the same disease process.

## 2. Case Presentation

**Figure 1:**
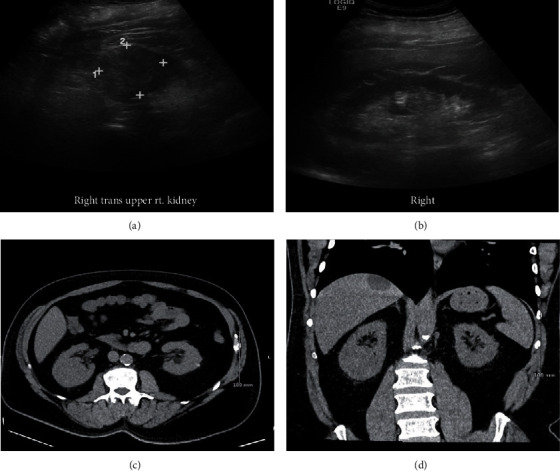
Ultrasound findings: transaxial (a) and long axis (b) views show the right kidney containing a solid, heterogeneous isoechoic mass in the midpole. CT abdomen without IV contrasts coronal (c) and axial (d) views, respectively: a solid isodense mass of the mid to inferior pole of the right kidney protrudes into the renal sinus. There is minimal deformity of the renal contour by the lesion. An incidental cystic lesion of the liver is seen.

**Figure 2:**
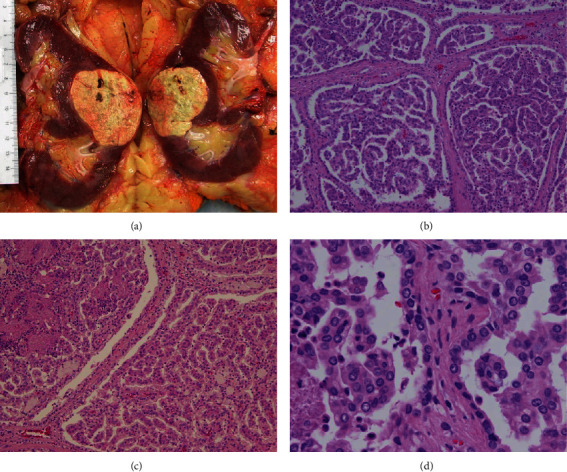
P-RCC gross and microscopic findings: (a) grossly, the tumor is less than 7 cm in diameter and confined to Gerota's fascia, making it a stage I tumor. (b) Papillary configuration of the architecture, with (c) necrosis intermittently throughout (100x magnification). (d) Pseudostratification with eosinophilic cytoplasm admixed with foamy histiocytes within a fibrovascular core (400x magnification). These are among the most characteristic features of type 2 papillary renal cell carcinoma.

**Figure 3:**
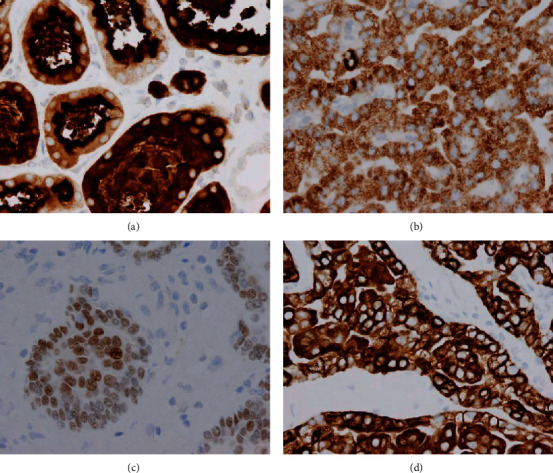
P-RCC immunohistochemistry: (a) CD10, (b) AMACR, and (c) PAX-8 are routinely positive in type II papillary renal cell carcinoma. (d) CK7 displays reactivity in the majority of type 1 papillary renal cell carcinoma, but is found in 20% of type 2 tumors as well (400x magnification).

The patient is a 58-year-old male with a past medical history of FSGS and CKD who had a midpole right kidney tumor discovered incidentally on ultrasound. He displayed none of the classic triad of RCC: flank pain, renal mass, or hematuria [1]. The ultrasound study described it as a “heterogenous isoechoic mass” (Figures 1(a) and 1(b)) after initially being called a “complex renal cyst”. A computerized tomography (CT) scan of the abdomen one week prior to surgery concurred and pointed out that despite being protruding into renal sinus, the renal contour was minimally deviated (Figures 1(c) and 1(d)). A radical nephrectomy was performed, and the patient recovered unremarkably. His blood creatinine level prior to surgery was 1.7 mg/dL and was last recorded as 2.7 mg/dL on postoperative day 3. The tumor was 5.5 × 4.5 × 4.0 cm in size, well circumscribed, and tan-yellow in appearance. The tumor abutted the fatty renal sinus and Gerota's fascia (Figure 2(a)). The tumor was confined to the renal parenchyma and did not invade any regional lymph nodes. The adrenal gland and rib removed with it were both unremarkable. Histological examination revealed a papillary configuration of pseudostratified cuboidal cells with high nuclear grade and eosinophilic cytoplasm (Figure 2(b)). Intrapapillary foamy macrophages were present in clusters. Tumor necrosis was variably present in different areas (Figure 2(c)). The tumor cell nucleoli were conspicuous and eosinophilic at 400x magnification and visible but not prominent at 100x magnification (Figure 2(d)). By immunohistochemistry, the tumor cells are positive for CD10, alpha-methyacyl-CoA racemase (AMACR), PAX-8, and CK7 (Figure 3). These features classified it as a type 2 P-RCC. A tumor staging of T1b was assigned. Additionally, FSGS, with less than 10% globally sclerosed glomeruli, was identified, along with interstitial fibrosis and mild arthrosclerosis. Significant comorbidities included FSGS, usual type, and CKD stage III, both occurring 1.5 years prior to the RCC diagnosis. It was also important that the patient admitted being a lifetime smoker and had a past medical history of chronic obstructive pulmonary disease (COPD), atrial fibrillation with pacemaker, and having a family history of breast cancer in several women on his mother's side of the family. While individually these comorbidities may not be indicative of the development of a malignancy, their combination with a nephrotic syndrome could be suggestive of further uncovered comorbidities, particularly malignancy. The finding of CKD III may be a significant precursor, since as many as 25% of RCC patients have had CKD present prior to nephrectomy [2].

**Figure 4:**
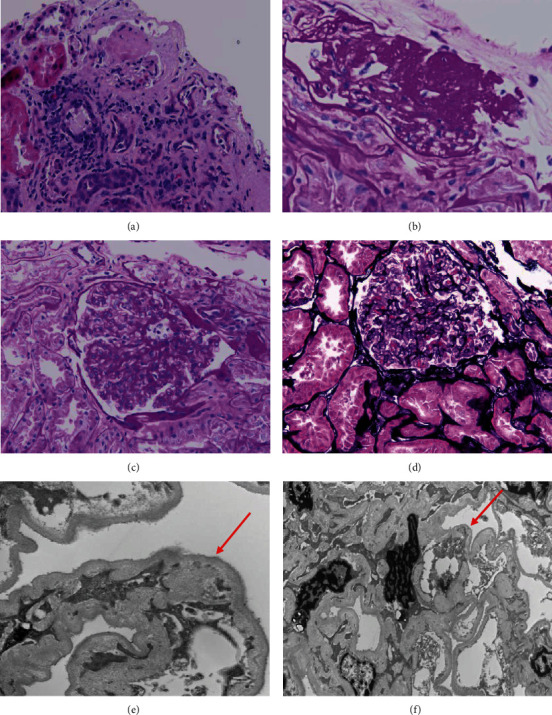
FSGS: (a) renal cortex with glomerulosclerosis and focal interstitial fibrosis, H&E stain (100x magnification). (b) Global glomerulosclerosis, PAS stain (200x magnification). Normal glomeruli, by definition, must coexist with the pathological process for the diagnosis of FSGS, PAS stain (c) and silver stain (d) (200x magnification). Electron microscopy: (e) foot process effacement, epithelial cell detachment from glomerular basement membrane, and capillary wrinkling (15,000x magnification). (f) Retraction of tufts and wrinkling of capillary walls (12,000x magnification).

In this patient, the FSGS diagnosis was made on a 1.2 × 0.5 × 0.2 cm kidney biopsy 1.5 years earlier. On light microscopy, segmental sclerosis and consolidation of capillary tufts were noted in several viable glomeruli (Figure 4(a)). Rare globally sclerosed glomeruli were also identified (Figure 4(b)). In the viable glomeruli, no crescent, proliferation of capillary cells, or necrosis of capillary tufts was identified (Figure 4(c)). No inflammatory cells or microthrombi were seen. On silver-stained sections, the open capillary loops showed thin and smooth capillary membranes, and no spikes or duplication of capillary membranes was identified (Figure 4(d)). Focal moderate interstitial fibrosis was present on trichrome-stained sections. There was no significant inflammatory infiltration in the interstitial compartment. The arterioles and small-sized arteries revealed mild fibrointimal thickening. There was no tissue submitted for immunofluorescence due to limited glomeruli that were present in the biopsy tissue. Electron microscopy examination confirmed FSGS with diffuse effacement of podocyte foot processes, epithelial cell detachment from glomerular basement membrane, and capillary wrinkling (Figure 4(e)). In most loops, the capillary loop basement membranes were uniform and of normal thickness. Retraction of tufts and wrinkling of capillary walls were noted (Figure 4(f)). There was no capillary loop hypercellularity, and no electron dense deposits were identified. The mesangial matrix was only mildly expanded, and no hypercellularity or electron dense deposits were present. No fibrils were identified in the renal tissue. These findings excluded immune complex disease and showed podocyte foot process effacement, consistent with usual type FSGS.

## 3. Discussion

Renal cell carcinoma has a propensity for presenting with a variety of paraneoplastic syndromes. These include, but are not limited to Cushing syndrome, leukemoid reaction, Stauffer syndrome, and polyneuromyopathy [3]. PG's are rare sets of symptoms that can present before, at the time of diagnosis, during remission, or at relapse of the various types of malignancies. Nephrotic syndrome is the most common PG. Pulmonary and GI tract malignancies and Hodgkin's lymphomas are the most likely malignancies to develop PG. These cancers tend to be associated with membranous nephropathy (MN) and minimal change disease (MCD) when PG is present [4]. When PG is associated with RCC, they are most commonly IgA nephropathy, MN, and rapidly progressive glomerulonephropathy. The FSGS seen in this patient is not even in the top 5 of the most common PGs in renal malignancies [3].

Specifically, concerning FSGS, it is the most common primary cause of nephrotic syndrome in adults. FSGS causes partial and segmental sclerosis of intermittent glomeruli, paired with nephrotic range proteinuria (>3.5 grams/24 hours). It can be caused by long standing hypertension, a comorbidity present in our patient. Additionally, the CKD III may be at least partially explained by the FSGS, which can progress to end-stage renal disease. The Columbia classification identifies five subtypes of FSGS. They are cellular, collapsing, perihilar, tip, and the most common, usual type/not otherwise specified [5]. The lattermost is present in this patient. With the incidence of FSGS more than doubling in the past two decades [6], a clinical presentation like that seen in our patient, of glomerulopathy, hypertension, CKD, and subsequent renal malignancy may become more common over time.

Type 2 P-RCCs represent 25% of P-RCCs, making it as common as type 1 P-RCC, both of which are less common than a mixture of type 1 and type 2 P-RCC at 50% [7]. Type 2 P-RCCs usually present at more advanced stages than type 1 and have a poorer prognosis. Early detection of warning signs for the development of this tumor type could be of great clinical significance.

There are two familial RCC syndromes that can have a papillary configuration. The first is hereditary papillary renal cell carcinoma. Hereditary P-RCC, an autosomal dominant disorder, is the result of an activating mutation of the c-MET oncogene at chromosome 7q31. Multifocal or bilateral P-RCC's are usually present, and the subtype is most often type 1 P-RCC [8]. The other hereditary syndrome that can present with P-RCC is hereditary leiomyomatosis and renal cell carcinoma syndrome (HLRCCS). Also, an autosomal dominant syndrome, HLRCCS, results from an inactivation mutation of fumarate hydratase, which is located on 1q42.3-q43. The resulting accumulation of intracellular fumarate leads to the inhibition of prolyl-hydroxylase domain-containing proteins and subsequently affecting stability of transcription factor hypoxia-induced factor 1 (HIF-1), and tumorigenesis follows. While the histology may mimic type 2 P-RCC, it is a distinct tumor entity [9].

There has been a reported case of FSGS, tip variant, diagnosed alongside the eosinophilic variant of clear cell RCC, following a MCD diagnosis one year prior [10]. Our patient had type 2 P-RCC following prior diagnosis of FSGS and CKD. While also an eosinophilic tumor, type 2 P-RCC is distinct from the previous case, and additionally, his FSGS was of usual type. Because P-RCC appears hypovascular on radiologic imaging due to extensive necrosis, it can be easily underdiagnosed on imaging, as it was in this case. Type 2 P-RCC typically presents at more advanced stages than type 1, making it more important to find clinical indicators of the impending disease. This patient's clinical and pathological features highlight the possibility of coexistence of renal comorbidities and renal malignancy. Due to the patient's family history of breast cancer and substance usage history, it cannot be conclusively determined that his FSGS and P-RCC are pathologically linked. It does, however, raise an important question about clinical presentations, as well as another question about how aggressively clinicians should approach screening nonneoplastic renal diseases for more concerning malignant comorbidities. Early detection is possibly the single most important prognostic factor, as 5-year survival ranges from 60 to 80% in stage I P-RCC compared to approximately 5% in stage IV [11]. Rarely, some cases of RCC will present at their sites of metastasis without any renal symptoms [12]. This further highlights the need for early detection via recognizing possible secondary symptomatology. Occurrences of PG are too rare to advocate adopting a screening protocol, even if multiple comorbidities and a nephrotic syndrome are present in conjunction. However, a case like this may highlight an underlying diagnosis when seemingly idiopathic glomerulopathy presents first. To our knowledge, FSGS as a preceding PG in type 2 P-RCC has not been documented before and must be approached with scrutiny, even in this case. It is important for clinicians to at least consider the possibility of malignancy if there is a higher index of suspicion, as this would drastically affect patient management.

## References

[1] Jayson M., Sanders H. (1998). Increased incidence of serendipitously discovered renal cell carcinoma. *Urology*.

[2] Hu S. L., Chang A., Perazella M. A. (2016). The nephrologist’s tumor: basic biology and management of renal cell carcinoma. *Journal of the American Society of Nephrology*.

[3] Lien Y.-H. H., Lai L.-W. (2011). Pathogenesis, diagnosis and management of paraneoplastic glomerulonephritis. *Nature Reviews Nephrology*.

[4] Bacchetta J., Juillard L., Cochat P., Droz J.-P. (2009). Paraneoplastic glomerular diseases and malignancies. *Critical Reviews in Oncology/Hematology*.

[5] D'Agati V. D., Fogo A. B., Bruijn J. A., Jennette J. C. (2004). Pathologic classification of focal segmental glomerulosclerosis: a working proposal. *American Journal of Kidney Diseases*.

[6] Vijayan M., Koshy P., Parthasarathy R., Mathew M., Abraham G. (2018). An unusual association of renal cell carcinoma and renal malakoplakia with focal segmental glomerulosclerosis in an elderly patient. *Indian Journal of Nephrology*.

[7] Chevarie-Davis M., Riazalhosseini Y., Arseneault M. (2014). The morphologic and immunohistochemical spectrum of papillary renal cell carcinoma: studying including 132 cases with pure type 1 and type 2 morphology as well as tumors with overlapping features. *The American Journal of Surgical Pathology*.

[8] Adeniran A. J., Shuch B., Humphrey P. A. (2015). Hereditary renal cell carcinoma syndromes: clinical, pathologic, and genetic features. *The American Journal of Surgical Pathology*.

[9] Chen Y. B., Brannon A. R., Toubaji A. (2014). Hereditary leiomyomatosis and renal cell carcinoma: syndrome-associated renal cancer: recognition of the syndrome by pathologic features and the utility of detecting aberrant succination by immunohistochemistry. *The American Journal of Surgical Pathology*.

[10] Al-Delfi F., Herrera G. A. (2015). Conventional and papillary renal cell carcinomas and focal segmental glomerulosclerosis in a nephrectomy. *Pathology Case Reviews*.

[11] Li P., Wong Y. N., Armstrong K. (2016). Survival among patients with advanced renal cell carcinoma in the pretargeted versus targeted therapy eras. *Cancer Medicine*.

[12] Mirza R., Ellsworth S., King J., Sangster G., Shi M. (2019). Cutaneous metastasis of renal cell carcinoma: fine needle aspiration provides rapid diagnosis. *Clinical Case Reports*.

